# DoGFinder: a software for the discovery and quantification of readthrough transcripts from RNA-seq

**DOI:** 10.1186/s12864-018-4983-4

**Published:** 2018-08-08

**Authors:** Yuval Wiesel, Niv Sabath, Reut Shalgi

**Affiliations:** Department of Biochemistry, Rappaport Faculty of Medicine, Technion–Israel Institute of Technology, Haifa, 31096 Israel

**Keywords:** Transcription, Transcriptional readthrough, Transcription regulation, Transcriptomics

## Abstract

**Background:**

Recent studies have described a widespread induction of transcriptional readthrough as a consequence of various stress conditions in mammalian cells. This novel phenomenon, initially identified from analysis of RNA-seq data, suggests intriguing new levels of gene expression regulation. However, the mechanism underlying naturally occurring transcriptional readthrough, as well as its regulatory consequences, still remain elusive. Furthermore, the readthrough response to stress has thus far not been investigated outside of mammalian species, and the occurrence of readthrough in many physiological and disease conditions remains to be explored.

**Results:**

To facilitate a wider investigation into transcriptional readthrough, we created the DoGFinder software package, for the streamlined identification and quantification of readthrough transcripts, also known as DoGs (Downstream of Gene-containing transcripts), from any RNA-seq dataset. Using DoGFinder, we explore the dependence of DoG discovery potential on RNA-seq library depth, and show that stress-induced readthrough induction discovery is robust to sequencing depth, and input parameter settings. We further demonstrate the use of the DoGFinder software package on a new publically available RNA-seq dataset, and discover DoG induction in human PME cells following hypoxia – a previously unknown readthrough inducing stress type.

**Conclusions:**

DoGFinder will enable users to explore, in a few simple steps, the readthrough phenomenon in any condition and organism.

DoGFinder is freely available at https://github.com/shalgilab/DoGFinder.

**Electronic supplementary material:**

The online version of this article (10.1186/s12864-018-4983-4) contains supplementary material, which is available to authorized users.

## Background

Readthrough transcripts, which we refer to as Downstream Of Gene containing transcripts, or DoGs, are a result of transcriptional readthrough events where RNA polymerases fail to terminate properly, and keep transcribing beyond the canonical transcription termination site. Recent studies have reported that various stress conditions lead to widespread readthrough transcription [1–4]. Using RNA-seq, we have identified massive induction of transcriptional readthrough in response to osmotic stress [1, 2], heat shock and oxidative stress [1] in mammalian cells, occurring in thousands of genes. In these conditions, thousands of DoGs, many extending for dozens of kilobases beyond gene ends, were highly induced, and interestingly, remained in the cell nucleus [1, 2]. In addition, readthrough induction has been demonstrated following HSV viral infection [3] and in renal carcinoma [4]. In our previous study, we have found several sequence and chromatin signatures that are characteristic of stress-induced DoGs [1]. Yet, the mechanism of readthrough induction and its role in gene expression and control remain elusive. Moreover, identification of naturally occurring readthrough in additional conditions, as well as in other non-mammalian organisms, is of great interest. Therefore, the ability to readily identify and quantify DoGs may provide new insights and understanding of the readthrough phenomenon and its relationship to gene expression regulation.

To that end, we developed DoGFinder, a python based software package that identifies DoGs and quantifies their expression levels from RNA-seq data. This package can be run on any RNA-seq dataset, single or paired-end, strand-specific or unstranded, polyA selected or non-selected, and can be used to identify readthrough transcripts in different conditions and different organisms.

## Implementation

DoGFinder was designed to identify and quantify the expression levels of readthrough transcripts, DoGs, for every annotated gene, given an RNA-seq bam file and gene annotation file(s). The DoGFinder package is written in python, and uses samtools [5] and bedtools [6], to search for continuous RNA-seq read density downstream of gene ends. Given any RNA-seq bam file, and one or more compatible gene annotation file(s), DoGFinder searches, downstream of every gene end in an input annotation, for a region of continuous read density (Additional file 1: Figure S1A). Candidate DoGs are identified by requiring a minimal coverage *minDoGCov* over a minimal initial length *minDoGLen* downstream of the 3′ end of every gene locus. Upon initial identification of candidate DoGs, DoGFinder elongates DoGs in overlapping running windows, until read coverage drops below *minDoGCov.* DoGFinder can be used to discover DoGs from any RNA-seq dataset, and produce DoG annotation files in bed format. It can further merge or intersect DoG annotation files (see below), and finally, quantify the expression levels of DoGs.

DoGFinder contains a number of functions as listed below:

*Get_loci_annotation*: In order to make sure that the DoG discovery procedure only considers non-genic reads, which are not related to the expression of different alternative isoforms or other non-coding RNAs, the *Get_loci_annotation* function (Additional file 1: Figure S1B) constructs a global loci annotation file in bed format, based on one or more user provided genome annotation gtf files, that are readily available for many sequenced genomes from the UCSC database [7], or other similar databases. For each gene locus, gene boundaries are set to be the most inclusive possible. Global loci annotation files for both human (hg19) and mouse (mm9) genomes, based on *Get_loci_annotation* run unifying RefSeq (refGene), UCSC (knownGene) and Ensembl (ensGene) annotations, are provided.

**Fig. 1:**
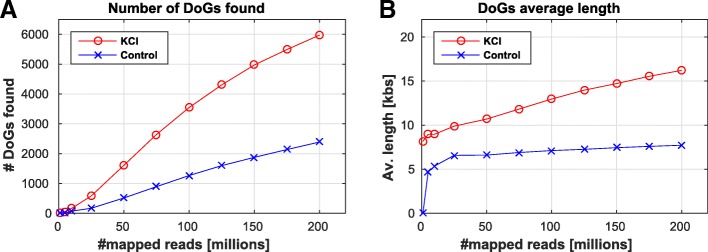
Osmotic stress DoG induction discovery using DoGFinder is robust to RNA-seq library depth. Performance test results of mouse NIH3T3 cells paired-end strand specific RNA-seq data before and after osmotic stress (2 h of KCl). Results show (**a**) the number of DoGs found in each condition, and (**b**) the DoG average length, as a function of library depth

*Pre_Process*: Before identifying DoGs, several pre-processing steps of the RNA-seq bam files are required (Additional file 1: Figure S1C). We note that the mapped RNA-seq bam files can be a result of any mapping program, however they should be mapped to a genome, rather than a transcriptome. First, using the RSeQC package [8], the library type is identified, and special strand processing is performed on paired-end libraries according to their strand type. Additionally, down-sampling of all the bam files is performed using samtools, in order to allow subsequent comparisons between DoGs from different samples. This is important, as sequencing depth can influence DoG discovery (see Results section below, Fig. 1), since the DoG identification process relies on continuous coverage criteria. This function is applied to all RNA-seq bam files in the dataset, and allows the use of multiple cores for parallel processing. *Pre_Process* can be run once for each dataset, and both pre-processed (in case of paired-end input datasets) and down-sampled bam files are kept, in order to save runtime for subsequent steps and to allow users to run *Get_DoGs* multiple times with different parameter settings.

*Get_DoGs*: Given a loci annotation bed file and a pre-processed RNA-seq bam file, the user can identify DoGs using the *Get_DoGs* function (Additional file 1: Figure S1A, D). The use of pre-processed bam files is required, especially in the case of paired-end data. First, the function removes all genic reads, and then identifies DoG candidates based on a minimal DoG length (*minDoGLen*), and minimal DoG coverage (*minDoGCov*). Then, the function elongates DoGs to find their putative endpoint. Importantly, in stranded libraries, strand information is used for coverage, as well as subsequent quantification. Additionally, DoG boundaries are limited by the location of the nearest 3′ neighboring gene in the genome, and genes with 3′ nearest neighbor closer than *minDoGLen* are discarded. When using stranded libraries, DoG boundary limitations are set in consideration with genes on the same strand, while in the case of unstranded libraries, neighboring genes are used to constrain DoG boundaries regardless of strand. *minDoGLen* and *minDoGCov* default parameters were assigned to be 4000 bases and 60% coverage respectively, which we found to be suitable for polyA-selected RNA-seq libraries (see below), which are the major library type generated. However, we note that stricter minimal length and coverage parameters could be considered when using non-polyA selected RNA-seq libraries, as DoGs have been shown to remain nuclear [1, 2], and non-polyA selected RNA-seq libraries are often enriched with nuclear RNA. The *Get_DoGs* function outputs an annotation file of the identified DoGs in bed format.

*Union_DoGs_annotation*: If several DoG annotation files are generated, e.g. for different replicates or treatments, one can use this function to create a single DoG annotation bed file, that merges all DoG annotations by choosing the most downstream coordinate as the end coordinate for all DoGs identified in one or more files.

*Common_DoGs_annotation*: If users wish to use DoGs that are common between several input samples, e.g. DoGs that are common to all biological replicates, *Common_DoGs_annotation* generates a single DoG annotation bed file from the intersection of several input DoG annotation files, similarly to *Union_DoGs_annotation*.

*Get_DoGs_rpkm*: Finally, given a bed format DoG annotation file and a pre-processed RNA-seq bam file, the *Get_DoGs_rpkm* function calculates DoG expression levels by a simple RPKM metric (Reads Per Killobase per Million mapped reads). Strand information is taken into consideration if the RNA-seq libraries are stranded. Its output is a csv format tab-delimited text file that contains the DoG annotation, DoG length and DoG RPKM values (Additional file 1: Figure S1E).

## Results

### DoGFinder demonstrates sensitivity and robustness of readthrough detection to sequencing depth and recapitulates readthrough identification from various inputs

To test the performance of DoGFinder, and assess its sensitivity to varying sequencing depths, we used our published nuclear-enriched rRNA-depleted strand specific paired-end RNA-seq data from mouse NIH3T3 cells that were exposed to osmotic stress (KCl, 2 h) [1]. We previously identified that stress both induces DoGs in numerous genes, and that DoGs get massively longer. We therefore now asked how many DoGs can be identified and what their average length was, as a function of library depth, using the minimal DoG length and minimal DoG coverage parameters from our published study, namely *minDoGLen* of 4500 bases and *minDoGCov* of 80%. We note that, as this dataset is enriched in nuclear fractions, and is non-polyA selected, we used stricter minimal length and coverage parameters than the default values, which are more suitable for polyA selected RNA-seq libraries (see below). We performed this test by first mapping the raw RNA-seq fastq files to the mouse genome using Tophat2 [9], and subsequently down-sampling the resulting bam files of untreated and osmotic stress (KCl) samples to various depths using samtools [5], and running DoGFinder.

We found that the number of DoGs is dependent on the library depth (Fig. 1). Hence, it is important to run the DoGFinder *Pre_Process* step to ensure DoG discovery is not biased due to different sequencing depths that may vary between different samples within the same dataset. The finding that osmotic stress (KCl) induces readthrough in more genes, however, was robust to sequencing depth, and at any given coverage, DoGFinder identified around 2.7 fold more DoGs in osmotic stress vs. untreated cells (Additional file 1: Figure S2). Interestingly, while the number of DoGs has not been saturated even at a library size of 200 M reads, DoG length has reached saturation already at 25 M read library depth, but only for the untreated cells. Indeed, transcriptional readthrough is considered rare in untreated cells, and these untreated DoGs probably represent natural termination events that occur more than 4.5 kb downstream of the annotated transcript end. The average length of these unstressed cell transcripts was ~ 7.5 kb past the gene end (Fig. 1b), which is in agreement with previous reports of some distant natural termination sites in mouse and human cells [10, 11].

To further substantiate the sensitivity of DoGFinder, we tested its capabilities in identification of DoGs from another readthrough inducing condition, HSV infection, using a different type of input data, namely 4sU-labeled newly synthesized RNA [3]. It has been previously reported that HSV infection of human fibroblasts induces transcriptional readthrough in thousands of genes, by examining reads mapped to the 5 kb region downstream to all human gene ends [3]. Nevertheless, the boundaries of these HSV-induced DoGs and their lengths have not been systematically explored. We therefore used several different initial parameter settings to test the robustness of DoGFinder in discovering the induction in the number of DoGs following HSV infection, and characterized their typical lengths. Using DoGFinder we found that thousands of DoGs are indeed induced at 7-8 h post HSV infection, compared to only hundreds identified in control conditions (Additional file 1: Figure S3A). Untreated cells showed readthrough lengths compatible with known transcription termination ends, around 7.5 kb on average, in agreement with our findings about mouse DoG ends in untreated cells in Fig. 1b. Moreover, DoGs got significantly longer post HSV infection, up to 19.8 kb on average over the different parameter settings (Additional file 1: Figure S3B). HSV-induced DoG were about 2.6 fold longer on average than in control condition, and this was robust to initial parameter settings (Additional file 1: Figure S3C). These results demonstrate the capabilities of DoGFinder in recapitulating and further expanding existing findings in previously identified readthrough-generating conditions.

### DoGFinder reveals induction of readthrough in response to hypoxia stress

**Fig. 2:**
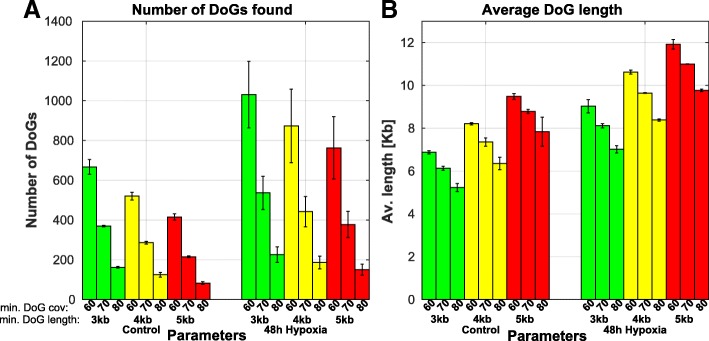
DoGFinder discovers readthrough induction following hypoxia in human cells. DoGFinder run on RNA-seq data of HPMECs before and after hypoxia stress (GEO accession GSE53510), with various initial parameter settings, show that hypoxia generates a larger number of DoGs (**a**) which are also getting longer (**b**). Bars represent means and error bars of DoGFinder runs on 2 or 3 replicates from control or hypoxia-treated cells, respectively (**a**), and over all DoG lengths (**b**), for various initial parameter settings

To illustrate the DoGFinder package usage on a new dataset, we used a publically available polyA selected single-end RNA-seq dataset of human pulmonary microvascular endothelial cells (HPMECs) before and after hypoxic stress (48 h of hypoxia, GEO Accession GSE53510). We mapped the raw RNA-seq data using Tophat2 [9] to the human genome to obtain bam files, and ran *Get_loci_annotation* on RefSeq, Ensembl and UCSC annotation gtf files of the human genome (build hg19) to generate the most inclusive gene annotation file. We subsequently ran *Pre_Process* on all the mapped bam files in order to get down-sampled bam files with similar depths, to allow subsequent comparison between untreated and hypoxic cells. We then asked whether hypoxia stress induces transcriptional readthrough, similarly to osmotic, oxidative and heat stress, and ran *Get_DoGs* with varying minimal DoG coverage and minimal DoG length parameter settings. Interestingly, we found that hypoxia stress induces readthrough transcription (Fig. 2). Both the number of DoGs and the DoG average length were higher in hypoxia treated compared to control cells for all minimal DoG coverage and minimal DoG length parameter combinations tested (Fig. 2).

Having found that readthrough induction in hypoxia is robust to parameter settings, we chose to proceed with a minimal DoG length of 4 kb and minimal DoG coverage of 60%. We used *Common_DoGs_annotation* to get the list of DoG sets common to all biological replicates within each treatment, and reassuringly, the overlaps were very high (Additional file 1: Figure S4); we found 508, 509 and 543 DoGs in the three control replicates, with 420 DoGs common to all three replicates (*p* < 10^− 323^, using a statistical test designed to test the significance of a multi-group intersections [12]), and 742 and 1004 DoGs in the two hypoxia replicates, with an overlap of 688 DoGs (*p* = 1.2*10^− 85^, using a hypergeometric *p*-value).

**Fig. 3:**
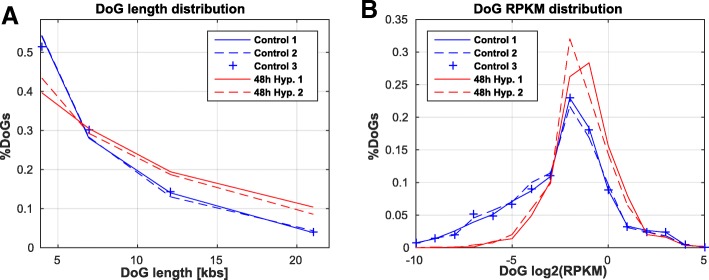
Hypoxia-induced DoGs are longer and more highly expressed. For a single parameter setting, minimal DoG length of 4000 bases and 60% coverage: **a** The DoG length distribution shows significantly longer DoGs in hypoxia (*p* = 6.7*10^− 4^, ranksum test) **b** DoG expression levels are significantly higher in hypoxia compared to control cells (*p* = 5.88*10^− 26^, ranksum test)

The DoG length distribution demonstrated that, similarly to other proteotoxic stress conditions, DoGs tend to get significantly longer under hypoxic stress (Fig. 3a, *p* = 6.7*10^− 4^ using ranksum test). We then used *Union_DoGs_annotation* to merge the lists of DoGs that were common to all replicates of a given condition, in order to obtain a unified DoG annotation file containing all DoGs identified in both control and hypoxia-treated cells. We finally ran *Get_DoGs_rpkm* on all bam files using the unified DoG annotations to calculate DoG expression levels across conditions. We found that, similarly to other stresses, DoG expression levels were significantly higher in hypoxia compared to the control cells (Fig. 3b, *p* = 5.88*10^− 26^ using ranksum test).

## Discussion

To the best of our knowledge, DoGFinder is the first tool specifically designed to identify and quantify readthrough transcription, whereas most standard RNA-seq quantification methods are based on existing annotations [13], and would thus not identify DoGs. Due to the abundance of RNA-seq in biomedical research, scientists can use DoGFinder to ask whether transcriptional readthrough occurs in any condition of interest, and identify the genes subject to readthrough, readthrough levels and lengths. Readthrough can often be identified from polyA-selected RNA-seq data, as we have previously shown for heat shock in mouse [1], and as demonstrated above for the human hypoxia dataset (Figs. 2 and 3), when using DoGFinder with looser *minDoGCov* and *minDoGLen* parameters. Nevertheless, non-polyA selected RNA-seq data often reveals a broader picture of DoG prevalence, length and expression.

DoGFinder can run on any RNA-seq data of any sequenced genome. However, the discovery of readthrough relies on the availability of good transcript annotations. Nevertheless, many less well-annotated sequenced genomes have basic annotations of CDSs (coding sequences), often based on homology. One possibility is that DoGFinder can be easily adapted, in poorly annotated genomes, to identify 3’ UTRs downstream of annotated CDSs. Further comparisons between different conditions, and more subsequent analyses will be required to delineate 3’ UTRs from readthrough transcripts in poorly annotated genomes, and those may be subjects for future software improvements.

In our previous comparative study on stress-induced transcriptional readthrough, we identified a total of 4852 DoGs induced in at least one of three stress conditions: heat shock, oxidative, and osmotic stress, in mouse NIH3T3 cells. Osmotic stress had the largest number of DoGs identified: over 92% of the entire set, suggesting that this number, representing about 60% of expressed genes in NIH3T3 cells, is close to the actual percentage of stress-induced DoGs in mammalian genomes. However, since these are three proteotoxic stress conditions, we wondered if comparison between osmotic stress DoGs and HSV infection DoGs would reveal a more elaborate picture. Comparing osmotic stress DoGs from human SK-N-BE(2) C cells [2] with HSV-induced DoGs from HFF cells [3] using DoGFinder with *minDoGLen* of 4000 bases and *minDoGCov* of 80% (DoGs common to both replicates in each condition) showed that 30% of the ~ 2200 DoGs discovered were unique to HSV compared to osmotic stress. Furthermore, out of the 641 genes associated with these HSV-exclusive DoGs, only a third were not expressed in the SK-N-BE(2) C cells. This suggests that different perturbations, stress conditions and cell types, combined with deep non polyA-selected RNA-seq, can reveal more previously-unidentified DoGs.

Naturally occurring readthrough transcription is of growing interest [14], and was found to be very widespread in several conditions. We previously suggested that some DoGs may function in antisense regulation [1], which has indeed been demonstrated by Muniz et al. [15]. We further showed that stress-induced DoGs harbor specific sequence and chromatin signatures [1], arguing for a potential role in maintenance of open chromatin. Nevertheless, the exact role of DoGs in regulation of gene expression is still unresolved. DoGFinder can now provide streamlined identification and quantification of readthrough transcripts from any RNA-seq data, which will facilitate further research into the causes and consequences of transcriptional readthrough.

## Conclusions

The DoGFinder software package, in a few simple steps, facilitates a flexible and streamlined identification and quantification of readthrough transcripts from any type of RNA-seq data, in any sequenced organism. DoGFinder is intended for a broad audience and does not require any bioinformatics knowledge or skills. The results of DoGFinder can provide: (1) new insights to existing RNA-seq datasets, as demonstrated for HPMECs subject to hypoxic stress; and (2) deeper characterization of existing readthrough conditions, as illustrated for the HSV-induced DoGs (Additional file 1: Figure S3). Newly identified readthrough transcripts can be further investigated to enhance our understanding of transcriptional regulation, stress responses, and the potential role of this new class of RNAs in physiology and disease. Additionally, the study of DoGs can shed new light on different transcription and polyadenylation regulatory programs that act to shape the transcriptome of various cell types and organisms under changing environments.

## Availability and requirements

Project name: DoGFinder.

Project home page: https://github.com/shalgilab/DoGFinder

Operating system(s): unix/linux/Mac.

Programming language: python.

Other requirements: Python2, Bedtools: version 2.20.0 or later, samtools, python with setuptools.

License: The package is freely available.

Any restrictions to use by non-academics: none.

## Additional files

Additional file 1: **Figure S1.** Illustrations of the DoGFinder package workflow and key functions. (A) DoGFinder workflow as detailed in the Implementation section, and the documentation on Github. (B-E) illustrations of the methodology and logic of the main DoGFinder functions. **Figure S2.** DoGFinder identification of osmotic stress-induced readthrough is robust to library depth. DoGFinder run on untreated and osmotic stress (KCl) treated NIH3T3 RNA-seq data [1] which were downsampled to varying depths using samtools. DoGFinder found on average 2.7 fold more DoGs in osmotic stress than in untreated cells, regardless of the library depth. **Figure S3.** DoGFinder recapitulates readthrough identification from various inputs. DoGFinder run on HSV-infected human cell 4sU-labeled RNA-seq dataset (7-8 h and control) from [3] illustrates the robustness of readthrough finding at 7-8 h post infection vs. control with respect to: (A) number of DoGs discovered (B) DoG length increase. (C). The ratio of average DoG lengths between HSV infected cells (7-8 h) and untreated cells is robust to DoGFinder initial parameter settings. **Figure S4.** High overlaps between biological replicates in control and hypoxia treated HPMECs DoG sets. Venn diagrams present the DoG overlaps between 3 and 2 biological replicates of control and hypoxia treated HPMECs. (A) We found 508, 509 and 543 DoGs in the control replicates, with an overlap of 420 DoGs (*p* < 10–300, using the hypergeometric *p*-value from [12]). (B) We found 742 and 1004 DoGs in the hypoxia replicates, with an overlap of 688 DoGs (*p* = 1.2*10–85, using a hypergeometric p-value). (PDF 778 kb)

## Abbreviations

DoGs: Downstream of gene-containing transcripts
HPMECs: Human pulmonary microvascular endothelial cells
RPKM: Reads Per kilobase per million mapped reads
