# Supplementary material for: DoGFinder: a software for the discovery and quantification of readthrough transcripts from RNA-seq
Source: BMC Genomics. 2018 Aug 8;19:597. doi: 10.1186/s12864-018-4983-4 (PMC6083495; doi:10.1186/s12864-018-4983-4)
Supplement: Supplementary file 1 — Figure S1. Illustrations of the DoGFinder package workflow and key functions. (A) DoGFinder workflow as detailed in the Implementation section, and the documentation on Github. (B-E) illustrations of the methodology and logic of the main DoGFinder functions. Figure S2. DoGFinder identification of osmotic stress-induced readthrough is robust to library depth. DoGFinder run on untreated and osmotic stress (KCl) treated NIH3T3 RNA-seq data [1] which were downsampled to varying depths using samtools. DoGFinder found on average 2.7 fold more DoGs in osmotic stress than in untreated cells, regardless of the library depth. Figure S3. DoGFinder recapitulates readthrough identification from various inputs. DoGFinder run on HSV-infected human cell 4sU-labeled RNA-seq dataset (7-8 h and control) from [3] illustrates the robustness of readthrough finding at 7-8 h post infection vs. control with respect to: (A) number of DoGs discovered (B) DoG length increase. (C). The ratio of average DoG lengths between HSV infected cells (7-8 h) and untreated cells is robust to DoGFinder initial parameter settings. Figure S4. High overlaps between biological replicates in control and hypoxia treated HPMECs DoG sets. Venn diagrams present the DoG overlaps between 3 and 2 biological replicates of control and hypoxia treated HPMECs. (A) We found 508, 509 and 543 DoGs in the control replicates, with an overlap of 420 DoGs (p < 10–300, using the hypergeometric p-value from [12]). (B) We found 742 and 1004 DoGs in the hypoxia replicates, with an overlap of 688 DoGs (p = 1.2*10–85, using a hypergeometric p-value). (PDF 778 kb) [file 12864_2018_4983_MOESM1_ESM.pdf]

Supplementary Information

DoGFinder: A software for the discovery and quantification of readthrough transcripts from RNA-seq.

Yuval Wiesel[1], Niv Sabath[1], Reut Shalgi[1,*]

[1] Department of Biochemistry, Rappaport Faculty of Medicine, Technion–Israel Institute of Technology, Haifa 31096, Israel

*To whom correspondence should be addressed: reutshalgi@technion.ac.il

A.

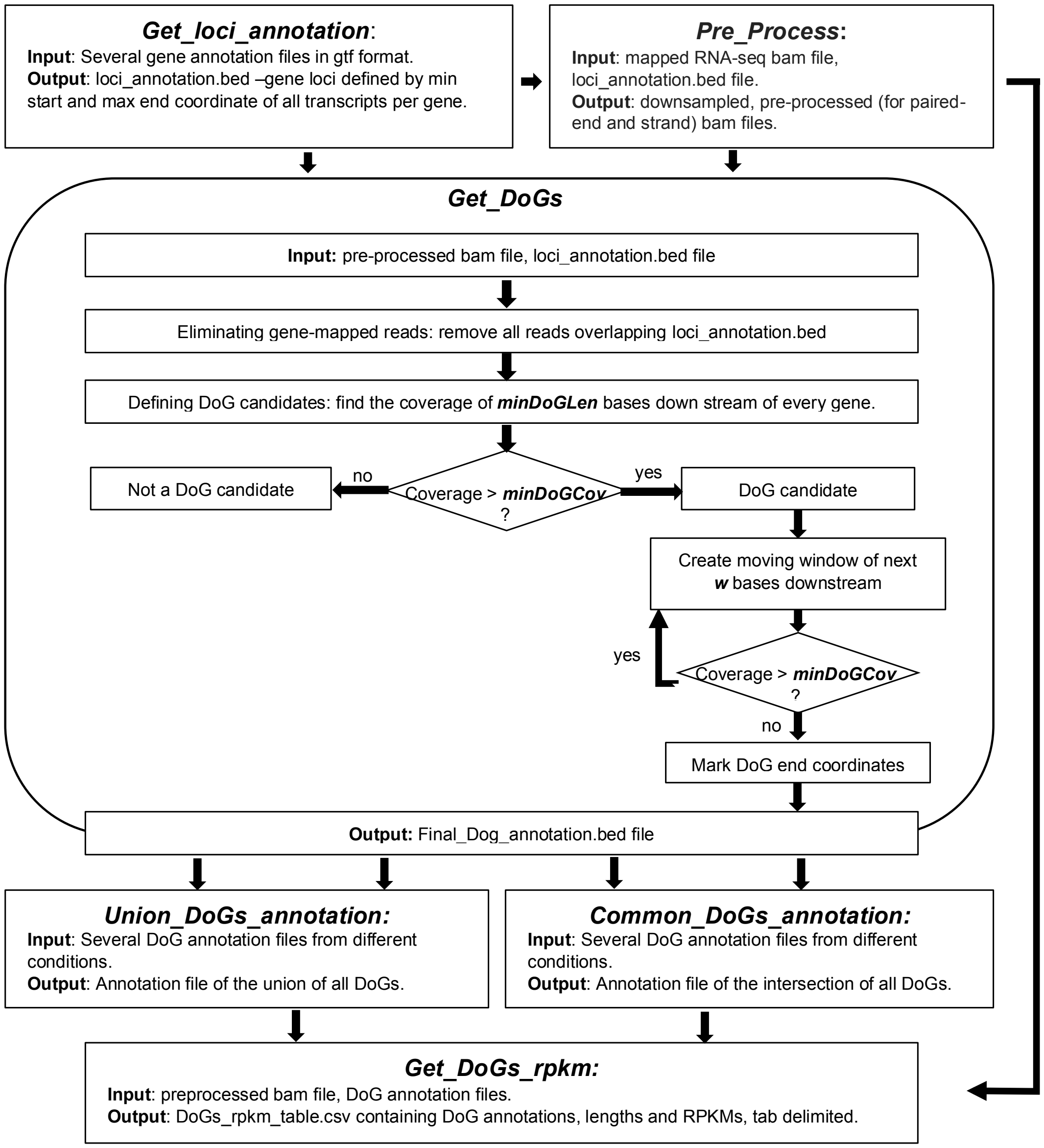
Get_loci_annotation:
Input: Several gene annotation files in gtf format.
Output: loci_annotation.bed –gene loci defined by min start and max end coordinate of all transcripts per gene.
Pre_Process:
Input: mapped RNA-seq bam file, loci_annotation.bed file.
Output: downsampled, pre-processed (for paired-end and strand) bam files.
Get_DoGs
Input: pre-processed bam file, loci_annotation.bed file
Eliminating gene-mapped reads: remove all reads overlapping loci_annotation.bed
Defining DoG candidates: find the coverage of minDoGLen bases down stream of every gene.
Not a DoG candidate
no
Coverage > minDoGCov ?
yes
DoG candidate
Create moving window of next w bases downstream
yes
Coverage > minDoGCov ?
no
Mark DoG end coordinates
Output: Final_Dog_annotation.bed file
Union_DoGs_annotation:
Input: Several DoG annotation files from different conditions.
Output: Annotation file of the union of all DoGs.
Common_DoGs_annotation:
Input: Several DoG annotation files from different conditions.
Output: Annotation file of the intersection of all DoGs.
Get_DoGs_rpkm:
Input: preprocessed bam file, DoG annotation files.
Output: DoGs_rpkm_table.csv containing DoG annotations, lengths and RPKMs, tab delimited.

B.

Create merged annotation file using ***Get_loci_annotation***:

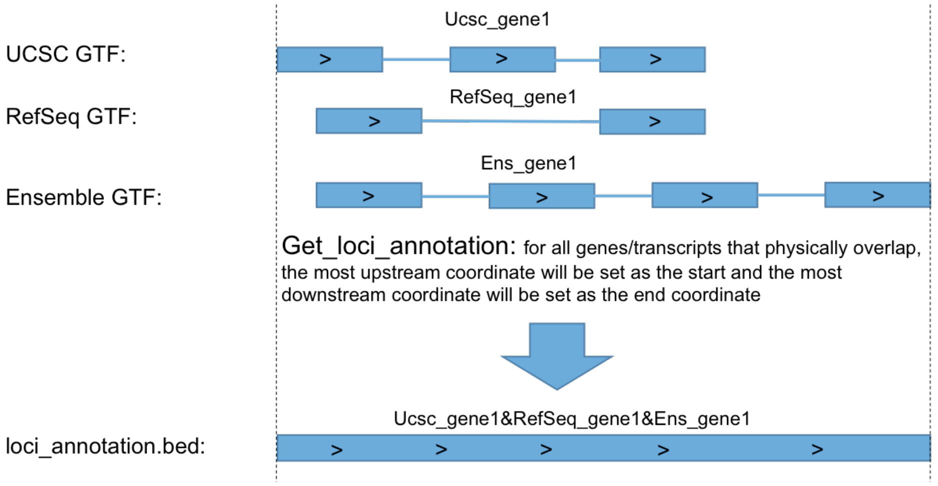

C.

Pre-processing steps of the RNA-seq bam files Using ***Pre_Process:***

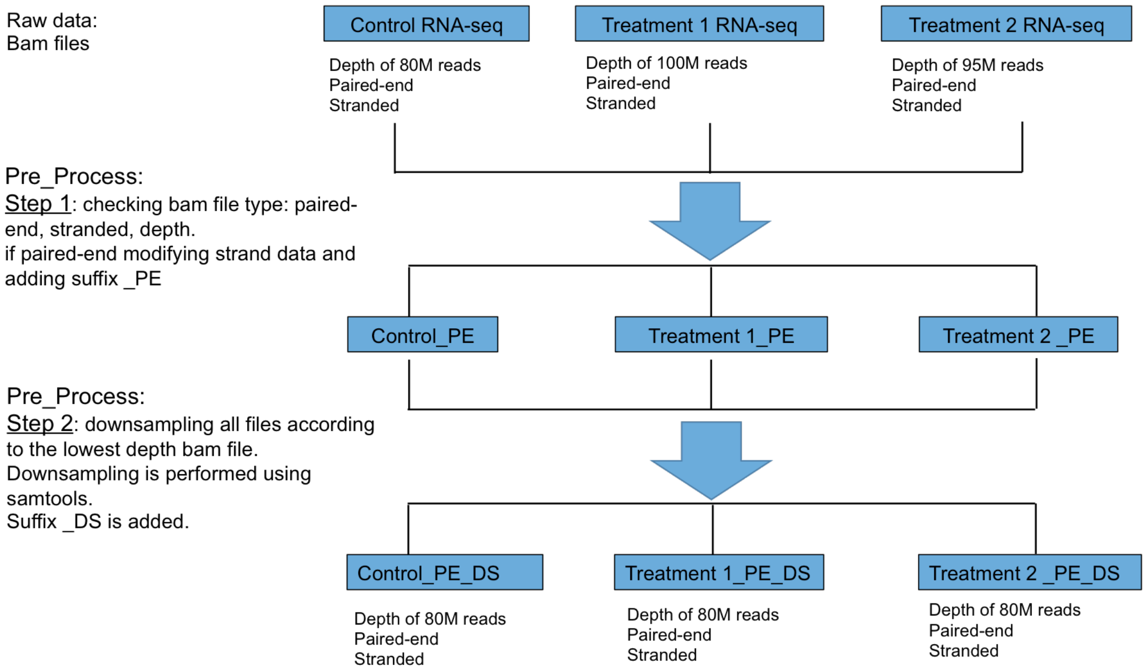

D. Find DoGs using ***Get_DoGs***:

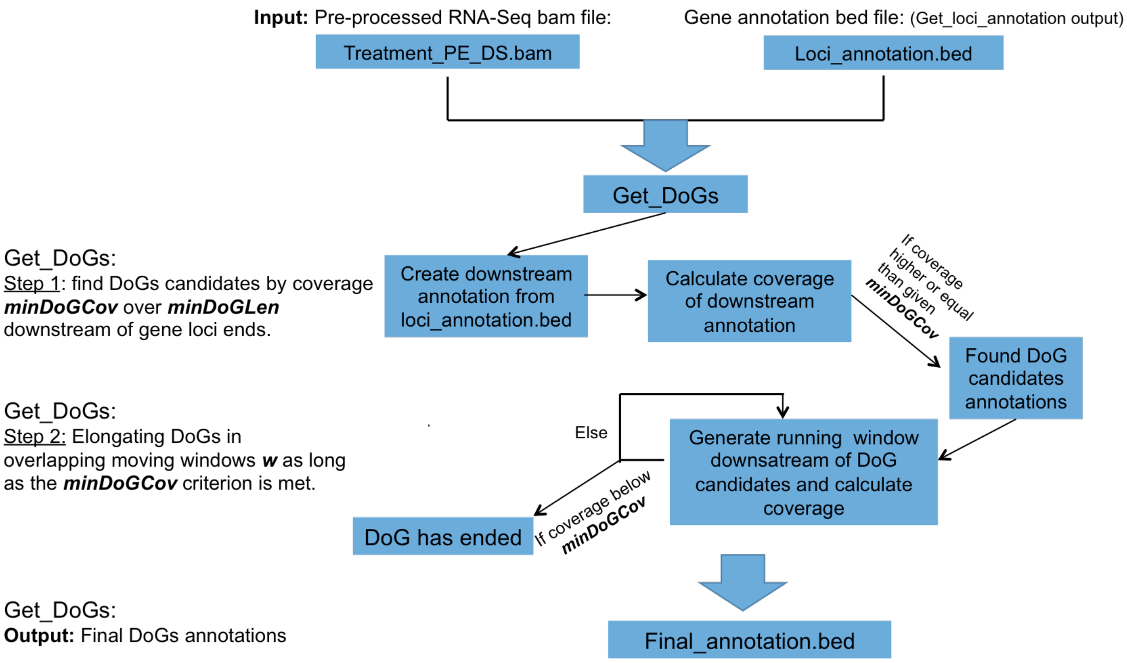

E. Calculate DoGs expression levels using ***Get_DoGs_RPKM***:

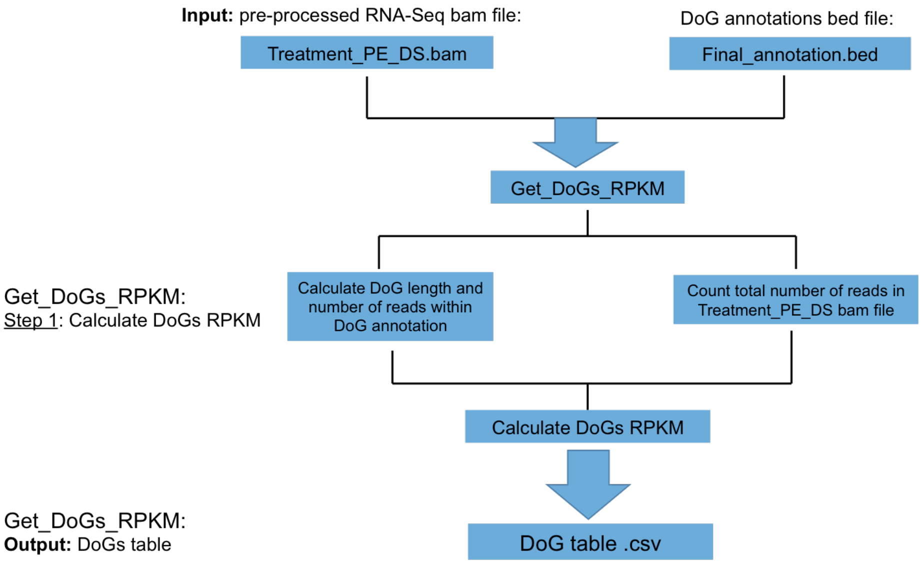

Figure S1: Illustrations of the DoGFinder package workflow, and key functions. (A) DoGFinder workflow as detailed in the Implementation section, and the documentation on Github. (B-E) illustrations of the methodology and logic of the main DoGFinder functions.

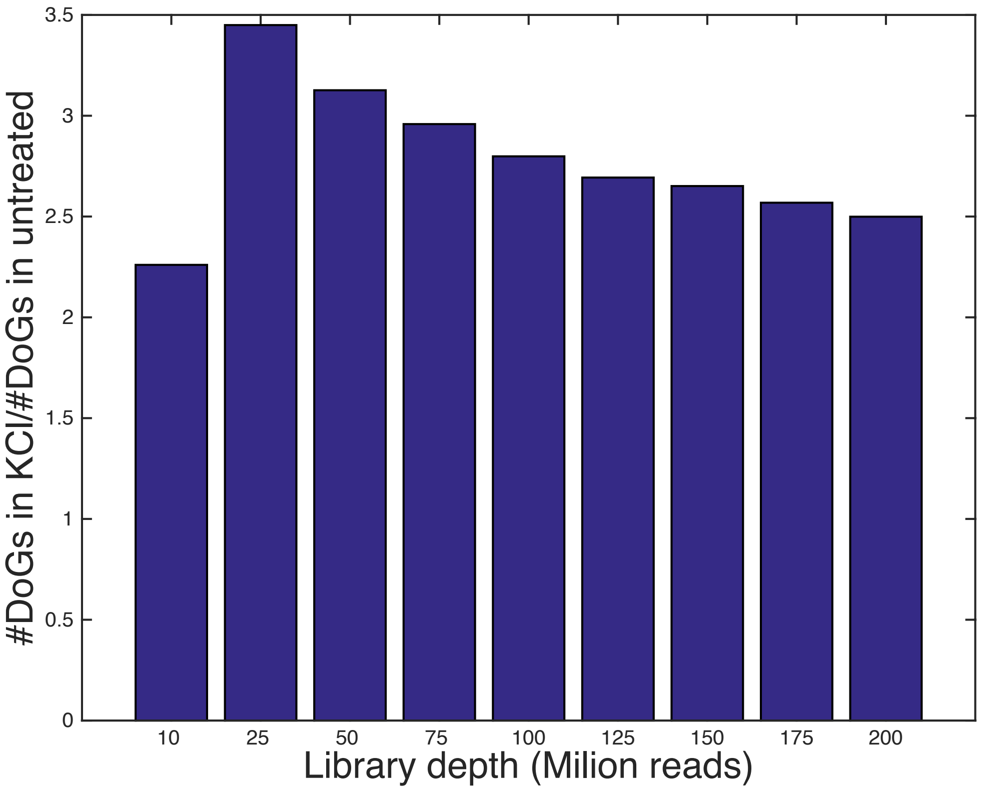

Figure S2: DoGFinder identification of osmotic stress-induced readthrough is robust to library depth.

DoGFinder run on untreated and osmotic stress (KCl) treated NIH3T3 RNA-seq data [1] which were downsampled to varying depths using samtools. DoGFinder found on average 2.7 fold more DoGs in osmotic stress than in untreated cells, regardless of the library depth.

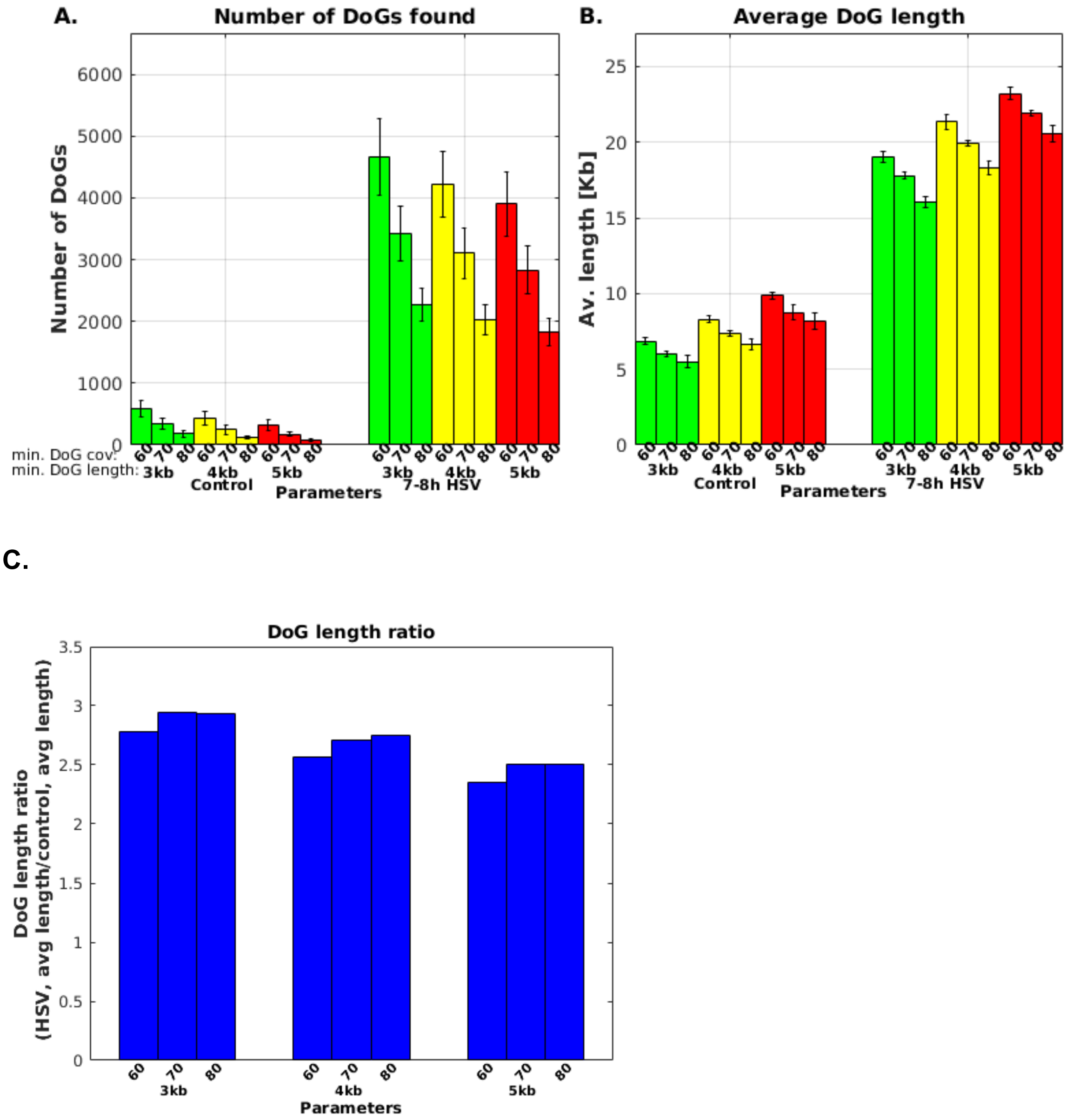

Figure S3: DoGFinder recapitulates readthrough identification from various inputs. DoGFinder run on HSV-infected human cell 4sU-labeled RNA-seq dataset (7-8h and control) from [2] illustrates the robustness of readthrough finding at 7-8h post infection vs. control with respect to: (A) number of DoGs discovered (B) DoG length increase. (C) The ratio of average DoG lengths between HSV infected cells (7-8h) and untreated cells is robust to DoGFinder initial parameter settings.

A.

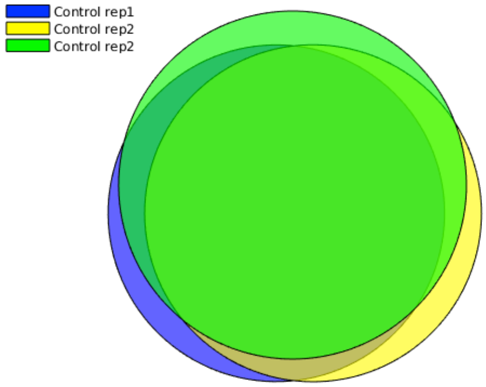

B.

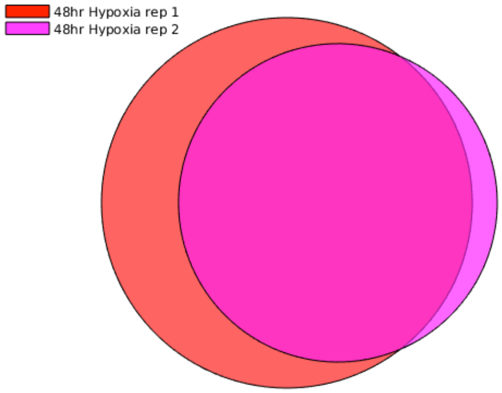

Figure S4: High overlaps between biological replicates in control and hypoxia treated HPMECs DoG sets.

Venn diagrams present the DoG overlaps between 3 and 2 biological replicates of control and hypoxia treated HPMECs. (A) We found 508, 509 and 543 DoGs in the control replicates, with an overlap of 420 DoGs ($p<10^{-300}$, using the hypergeometric p-value from [3]). (B) We found 742 and 1004 DoGs in the hypoxia replicates, with an overlap of 688 DoGs (p=1.2*10-85, using a hypergeometric p-value).

References:

1. Vilborg A, Sabath N, Wiesel Y, Nathans J, Levy-Adam F, Yario TA, Steitz JA, Shalgi R: **Comparative analysis reveals genomic features of stress-induced transcriptional readthrough**. *Proc Natl Acad Sci U S A* 2017, **114**(40):E8362-E8371.
2. Rutkowski AJ, Erhard F, L'Hernault A, Bonfert T, Schilhabel M, Crump C, Rosenstiel P, Efstathiou S, Zimmer R, Friedel CC *et al*: **Widespread disruption of host transcription termination in HSV-1 infection**. *Nat Commun* 2015, **6**:7126.
3. Wang M, Zhao Y, Zhang B: **Efficient Test and Visualization of Multi-Set Intersections**. *Sci Rep* 2015, **5**:16923.
